# Gain enhancement of BiCMOS on-chip sub-THz antennas by mean of meta-cells

**DOI:** 10.1038/s41598-022-07902-0

**Published:** 2022-03-10

**Authors:** Matteo Stocchi, Zhibo Cao, Christopher Hardly Joseph, Thomas Voss, Davide Mencarelli, Luca Pierantoni, Canan Baristiran Kaynak, Joachim Hebeler, Thomas Zwick, Matthias Wietstruck, Mehmet Kaynak

**Affiliations:** 1 IHP Microelectronics, Im Technologiepark 25, 15236 Frankfurt (Oder), Germany; 2 Universitá Politecnica delle Marche, 60131 Ancona, Italy; 3 Istituto Nazionale di Fisica Nucleare (INFN) - Laboratori Nazionali di Frascati (LNF), Via E. Fermi, Frascati, Rome Italy; 4 IHE, Karlsruher Institute for Technology, Karlsruhe, Germany

**Keywords:** Electrical and electronic engineering, Computational science

## Abstract

A MM-loaded sub-THz on-chip antenna with a narrow beamwidth, 9 dB gain and a simulated peak efficiency of 76% at the center frequency of 300 GHz is presented. By surrounding the antenna with a single MM-cell ring defined solely on the top metal of the back-end of line, an efficient suppression of the surface waves is obtained. The on-chip antenna has been designed using IHPs 130 nm SiGe BiCMOS technology with a 7-layer metallization stack, combined with the local backside etching process aimed to creating an air cavity which is then terminated by a reflective plane. By comparing the measured MM-loaded antenna performances to its non-MM-loaded counterpart, an enhanced integrity of the main lobe due to the MM-cells shielding effect can be observed. An excellent agreement between the simulated and measured performances has been found, which makes the MM-loaded antennas a valid alternative for the upcoming next-generation sub-THz transceivers.

## Introduction

The sub-THz band communication has the advantages of high data volume and wide bandwidth, picturing great opportunities for future applications. The concepts and associated hardware demonstrations have been published in recent years^1–3^. However the high propagation loss has always been the pain point of this technology^4,5^. The development of a high gain, large bandwidth sub-THz antenna is, therefore, the key to unlock its advantages. On the other hand, the concept of on-chip antennas fits well to the sub-THz application by having high integration levels, low profiles and low costs of fabrication. Yet the antennas have to be planar and adhere to semiconductor design rules. The patch antennas, quasi-Yagi antennas and dipole antennas are most typical configurations used in printed fabrications. Among them, the patch antennas are usually featured with narrow bandwidths of only a few percentage^6,7^. Besides, the chip back-end dielectrics are usually tens of micrometer thick, not only limiting the bandwidth but making the on-chip patch efficiency low as well^8,9^. And compared with the quasi-Yagi and dipole antennas, the bow tie (modified dipole) antenna features with an even wider bandwidth^10^. To take full advantage of the THz band and aim for a high throughput, a bow tie antenna operating at 300 GHz is designed and fabricated to demonstrate our ideas. However, the on-chip antennas usually suffer from low gains^11^ due to the lossy silicon substrate and high frequency (the demand for large bandwidth leads to the choice of antennas with an intrinsic wide bandwidth but low gain such as dipoles). Many efforts have been made to increase its gain, such as thinning the substrate^12^, using high resistivity substrates^13^, using localized backside etching (LBE) to remove the substrate^14^ and using antenna arrays^15,16^. In recent years, the metamaterial concepts start to be exploited to enhance antenna performances, such as improving the gains^17,18^, shrinking the antenna sizes^19^, widening the bandwidths^20^, etc. Inspired by the metamaterial concepts, a meta-material cell (MM-Cell) ring is placed around the antenna to improve the antenna gain, thus mitigating the high propagation loss in sub-THz band. By avoiding using antenna arrays, which is usually area-consuming and thus costly especially in semiconductor industries, the size of the MM-cell antenna is only 2-3 times of a regular antenna size. Yet such a simple ring of MM-Cells are able to push the gain by 2 dBi in addition to the use of LBE without undermining its wide bandwidth. Such a result demonstrates an approach to achieve high gain, reduced beamwidth of sub-THz on-chip antennas, paving the way for sub-THz communication applications.

## Metamaterial unit cell design

**Figure 1:**
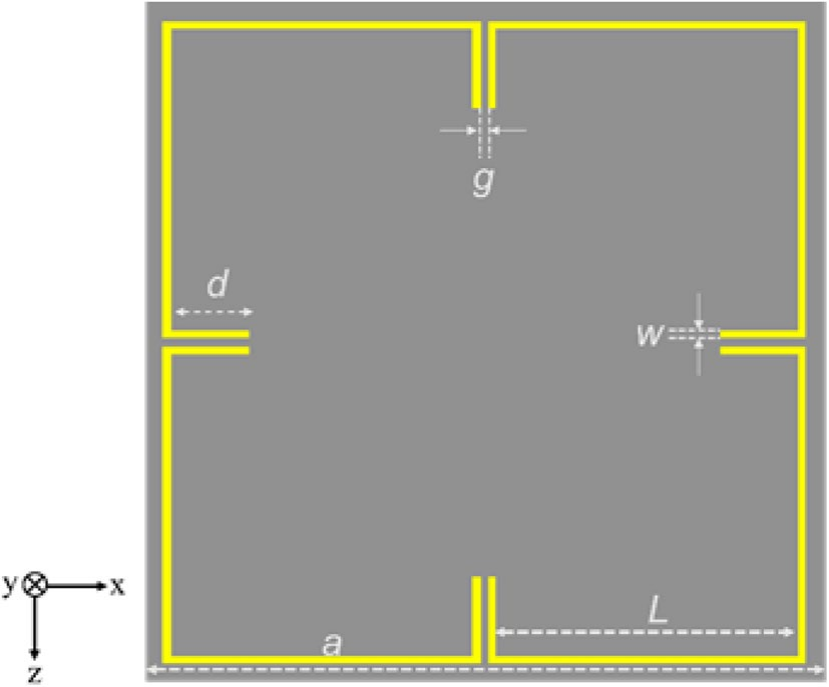
Geometry of the proposed metamaterial unit cell.

The novel metamaterial (MM) unit cell is designed by simplifying the conventional Split Ring Resonator (SRR) geometry. The designed metamaterial unit cell is illustrated in Fig  1. The MM unit cell is consisting of four distinct square like shaped loop rings with a capacitive split gap. The arm length (d) of these rings can be tailored to tune the desired resonance frequency. The slit gaps and the rings are arranged in a way to have an axis-symmetric structure. Several metamaterials embedded metasurfaces have been studied to enhance the on-chip antennas in terms of bandwidth and gain^21–23^. Some of those designs are requires complicated fabrication processes. The proposed metamaterial structure is easier to integrate and fabricate along with the antenna on chip. This design behaves similar like the conventional SRR where the stop band behaviour occurs mainly due to the inductance in the line and the capacitance in the split gaps. This geometry is simple compared to the concentric split ring resonators.

**Figure 2:**
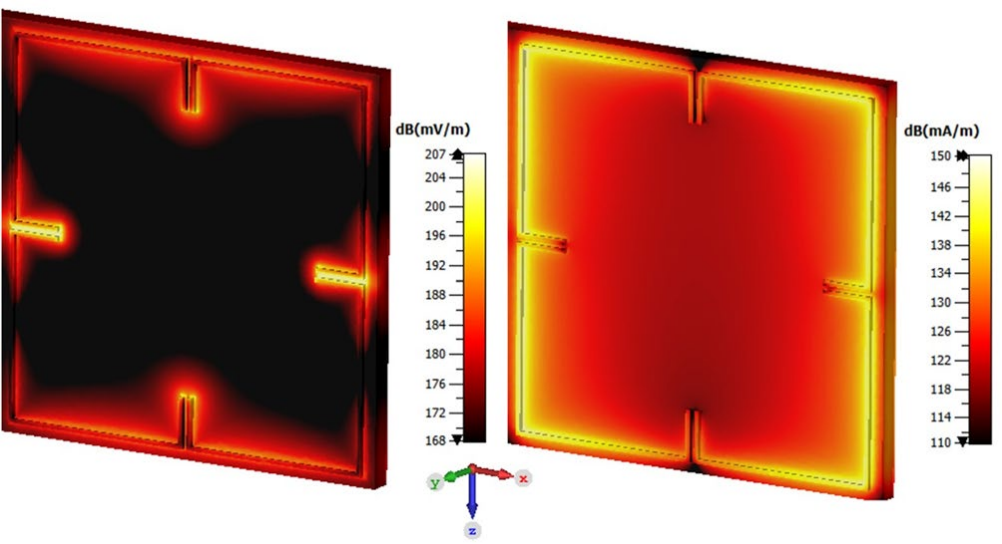
E-field and H-field distribution at 300 GHz. Figure created with COMSOL Multiphysics 5.5 (https://www.comsol.com/).

**Figure 3:**
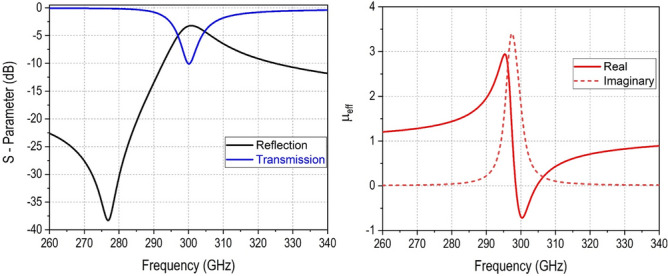
Reflection and transmission coefficients of the MM cell, real and imaginary parts of the estimated effective permeability.

The design parameters are chosen to have a resonance frequency at 300 GHz. The length and width of the unit cell is (a) 177 µm. The length of the arms of the split gaps (d) is 20.6 µm. The length of the rings (L) is 78.5 µm and the width of the rings (w) as well as the split gaps (g) are fixed as 2 µm. The MM cell is placed inside the 14.69 m thick SiO2 layer deposited on a silicon substrate which has a thickness of 130 $\mathrm {\mu }$m. The MM cell metallization is 3 µm and placed inside the SiO2 layer at 10.19 µm from the surface of the substrate. The electrical conductivity of the silicon substrate is considered as 2 S/m for the full wave simulation. The EM full wave simulation is performed in the frequency domain solver of the COMSOL Multiphysics 5.5 software. The unit cell boundary condition with a floquet port is considered to model the MM cell performance. The structure is excited by the incident plane wave (TE00 and TM00 modes) in the Z direction and exhibited a stop band at 300 GHz. The design has undergone several optimization steps by means of adjusting the design parameters to achieve a resonance at 300 GHz. Figure 2 shows the E-field and the H-field strength at the resonance frequency. The surface current circulates in the line and accumulates at the gaps resulting an enhanced electric field strength at the gaps and the enhanced magnetic field strength in the line is observed. The reflection and transmission characteristics of the MM cell obtained from the simulation is presented in Fig  3. A stop band at 300 GHz is observed. The magnitude of the $\mathrm {S_{11}}$ and $\mathrm {S_{21}}$ are − 3.2 dB and − 10.12 dB, respectively. As the structure is symmetric, both $\mathrm {S_{11}}$ and $\mathrm {S_{22}}$ are having similar values. The effective parameters of the metamaterial is retrieved from the scattering parameters obtained from the simulation using a method proposed in the work^24^.

A Matlab code has been developed for the retrieval of the effective material properties. The estimated effective permeability is shown in Fig  3. The real part of the permeability goes to negative at the resonance frequency as expected. This shows the resonance occurs purely due to the magnetic response of the designed MM cell similar to the conventional SRR. This confirms this design as a single negative metamaterial, and this helps in suppressing the surface waves produced in the substrate which results in enhancing the radiation gain^25^.

## Metamaterial antenna design

A square-like shape has been chosen for the test field (so that only one parameter is to be optimized), and the radiating element has been placed in the middle of it. Even though this is not, generally, the case when it comes to real chip designs, where usually the antenna is placed on the side of the chip, the selected geometry is sufficient for validating the MM-enhancing concept. In order to guarantee an acceptable radiation pattern, the LBE (Local Backside Etching) technology has been used on a bow-tie antenna printed on IHPs 130 nm high performance SiGe BiCMOS BEOL metallization stack, adopting the same approach used in^26^. For what concerns the antenna, a standard bow-tie has been chosen, so that good performances in band can be achieved. In any case, a similar approach can be used even for folded dipoles. A point of particular importance is represented by the characteristics of the glue that is supposed to stick together the die and the board (which, according to the design, must be metalized on top). When tuning the substrate thickness for maximizing the antenna efficiency, the additional thickness of the glue must be taken into account (if to operate at hundreds of GHz, the electromagnetic field is sensitive to variations of few µm). Even though the glue thickness is not usually taken into account when designing a generic antenna on-chip, in this context even a slight shifting of the antenna resonating frequency would be problematic given the fact that we want it to be the same as the MM-cells resonating frequency. For such matter, the following technological specifications have been followed; glue thickness between 5 and 10 µm, $\mathrm {\varepsilon _{r}}$ = 3 and $\mathrm {\delta }$ = 0.03.

**Figure 4:**
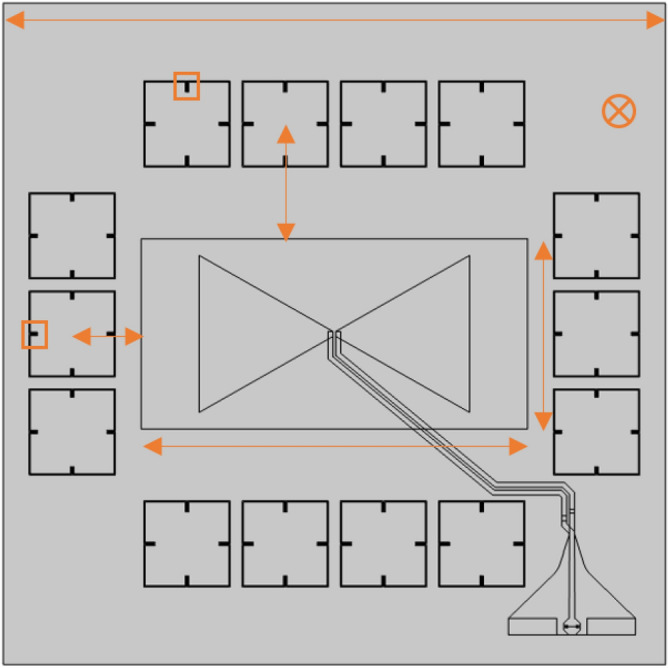
Snapshot of the simulated MM-loaded antenna with highlighted variables for the optimization. Figure created with COMSOL Multiphysics 5.5 (https://www.comsol.com/).

The test field area of 1.44 mm/sq has not been chosen casually. Given the rather limited area in which the LBE can be applied, the chip dimensions still critically affect the antenna performances as the substrate modes are attenuated but not totally canceled. For this reason, a chip size was chosen whose substrate modes did not destructively interfere with the bow-tie fundamental mode. According to Fig.  4, four lines of MM-cells have been placed around the antenna on-chip: two in the horizontal plane and other two in the longitudinal one. With respect to other MM-enhanced antenna designs^17,27^, it can be noted that the proposed configuration only consider a very limited numbers of resonators. It has been chosen to do so in order to minimize the area occupation of the radiating element as a whole (e.g. antenna + MM-cells), so that the proposed design can also be used in the next generation commercial applications. For what concerns the way the MM-cells responds to the incident field generated by the radiating element, it has been observed that the two lines placed on top and on the bottom of the antenna are more responsive in terms of resonance intensity. This is due to the different polarization of the E-field: according to the very nature of the resonating cell, their strongest resonance occurs when the polarization of the incident field is parallel, and if to consider the radiation pattern of a bow-tie antenna, such polarization is found along the y-axis. In accordance to that, the feeding line of the antenna on-chip has been designed so that the EMI between the latter and the bottom row of the MM-cells is minimized, even though a minimal effect is in any case to be expected. In general, for each of the six configurations that have been designed, the best antenna on-chip setup (in terms of maximum gain and directivity) has been obtained by sweeping a total of eight variables, namely:

- The chip side length,
- The distance between the center of the chip and the center of the two MM-cells line placed on the bottom and on top,
- The distance between the center of the test field and the center of the two MM-cells line placed on the right and on the left,
- The width of the LBE region,
- The height of the LBE region,
- The inset d of the MM-cells of the top and bottom lines,
- The inset d of the MM-cells of the right and left lines,
- The substrate thickness.

**Figure 5:**
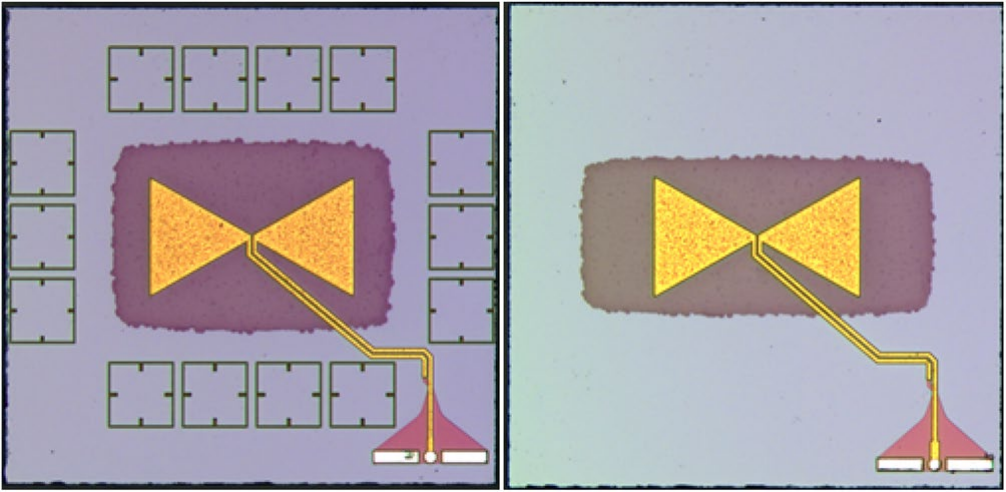
The fabricated MM-loaded and non-MM-loaded antennas.

Given the high number of variables, a specific strategy was needed for both the simulation setup and the meshing. For what concerns the simulation setup, two different sets of models have been produced: one used in the gain maximization phase, where the feeding line is neglected and a lumped port connects the two bow-tie terminals, and the complete one of Fig.  4, needed for matching purposes. Focusing on the model used in the gain optimization phase, the simulation time has been drastically reduced by exploiting the two symmetries of both the E- and H-planes. Switching to the mesh issue, the most critical point is represented by the different scales in play, e.g. by the sub-um thicknesses of the BEOL layers compared to the millimeter size of the chip. As a consequence, the standard tetrahedral approach would inexorably fails as the dimension of the tetrahedrons will be defined by the thin layers of the chip stack, and this leads on having a density of elements much higher than the required one in the transverse dimension^28^. The problem was solved by adopting a sweep-like approach. By meshing the transverse planes of the various domains with a triangular elements, the 2D-meshing is done in a rather relaxed way as the triangles are unconditioned by the limited depth of the layer. The boundary mesh is then swept along the z-direction, resulting in a mesh element that is both as extended as possible in the transverse dimension and as thin as needed in the longitudinal one. Figure 5 shows two samples of the fabricated antennas.

## Experimental setup

**Figure 6:**
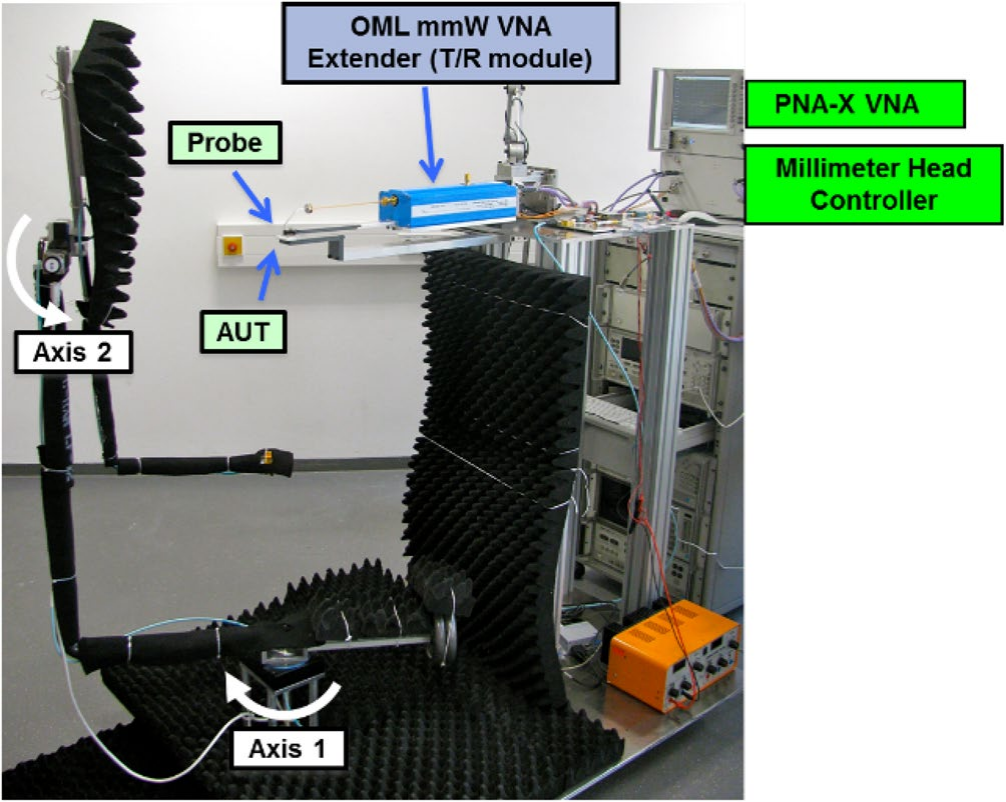
The used probe based free-space measurement setup.

To measure the antennas the probe based free-space measurement setup shown in Fig  6 was used.

Employing OML frequency extender, the antenna under test (AUT) can be measured from 220 GHz to 330 GHz with a 2-port VNA. Formfactor Infinity Waveguide GSG probes mounted directly to the extender contact the AUT. The setup employs two rotary stages to move a reference horn connected to a sub-harmonic mixer in a sphere around the AUT. Mechanical and electrical restrictions limit the coverable sphere surface. Using the three-antenna gain measurement technique both E- and H- antenna gain is measured. Further, the antennas impedance match is captured through the frequency extender, calibrated by 1-port SOL calibration.

## Results and discussion

**Figure 7:**
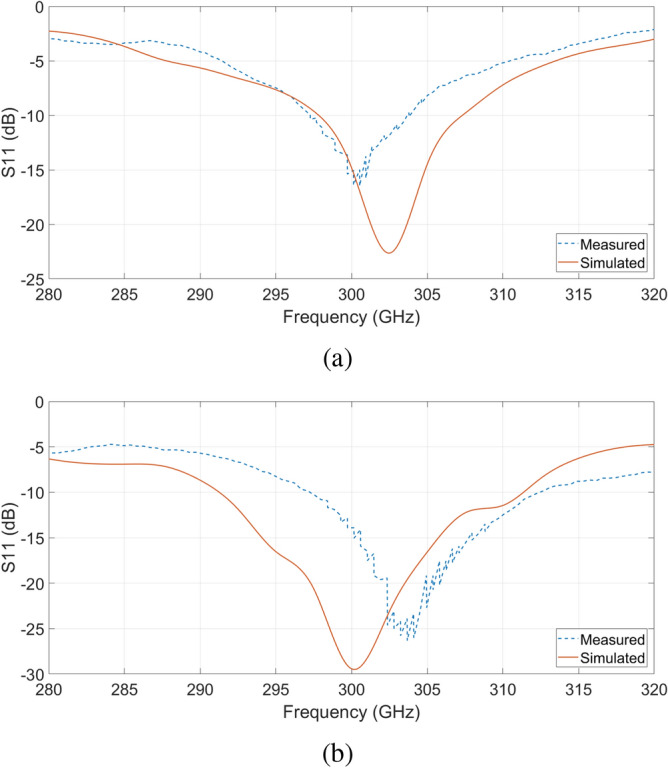
Measured and simulated reflection coefficients of the non-MM-loaded and MM-loaded antennas.

Figure 7 compares the reflection coefficients of the non-MM-loaded and the MM-loaded antennas, both simulated and measured. Although both of the resonance fall at 300 GHz as predicted by the simulations, one can notice a considerable increase in the bandwidth of the MM-loaded antenna, which gains approximately 10 GHz over the non-MM-loaded one. This improvement is not due to the MM-cells but rather to the different dimensions of the LBE areas of the two antennas under analysis. As this work is focused on the gain enhancement due to the surface wave suppression effect given by the MM-cells, the LBE area (together with all the other variables listed in the previous section) has been chosen in order to provide the highest gain for both the antennas rather than the largest bandwidth.

**Figure 8:**
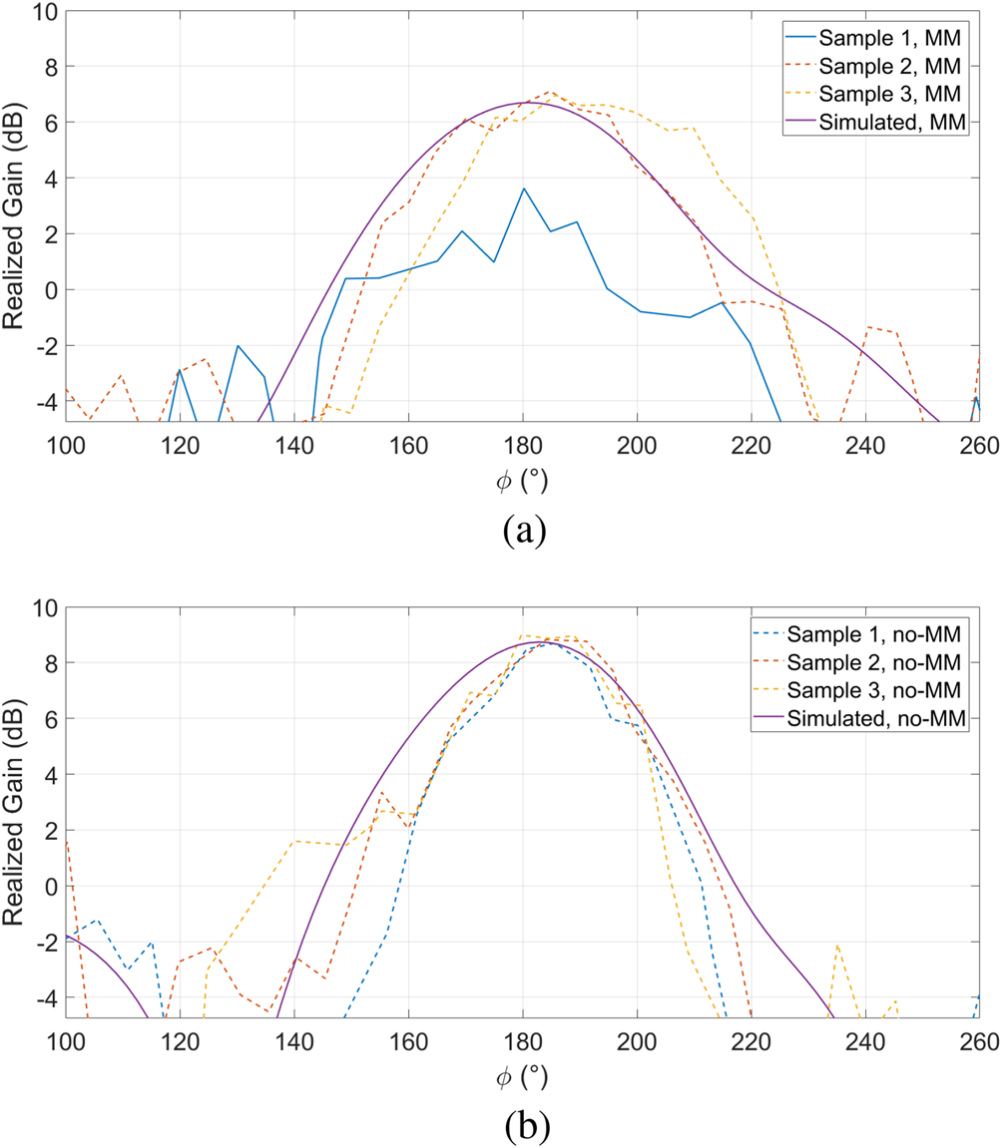
Measured and simulated radiation pattern for $\mathrm {\Theta }$ = 0$^{\circ }$ and $\mathrm {\phi }$ = 180$^{\circ }$ at 305 GHz of the non-MM-loaded and MM-loaded antennas.

Figure 8 compares the radiation patters (both simulated and measured) of the non-MM-loaded and the MM-loaded antennas at the frequency of 305 GHz, while also investigating the reliability of the measurements by considering different samples of the same antennas. One can immediately notice a net gain enhancement of 2 dB thanks to the presence of the MM-cells, together with a reduction of the beamwidth and a consequent increase of the directivity. Furthermore, it seems that the MM-enhanced antennas better preserve the integrity of their radiation patterns. In physical terms, such characteristic can be traced back, once again, to the MM-cells: as the latter efficiently suppress the surface waves, the antenna automatically become more isolated from the outer region of the chip, leading to a less relevant impact of the process imperfections (mainly due to the dicing-related cracks along the chip perimeter) on the antenna performances. That said, this can’t in any way be defined as a general rule given the fact that only three samples for each kind of antenna have been analyzed.

**Figure 9:**
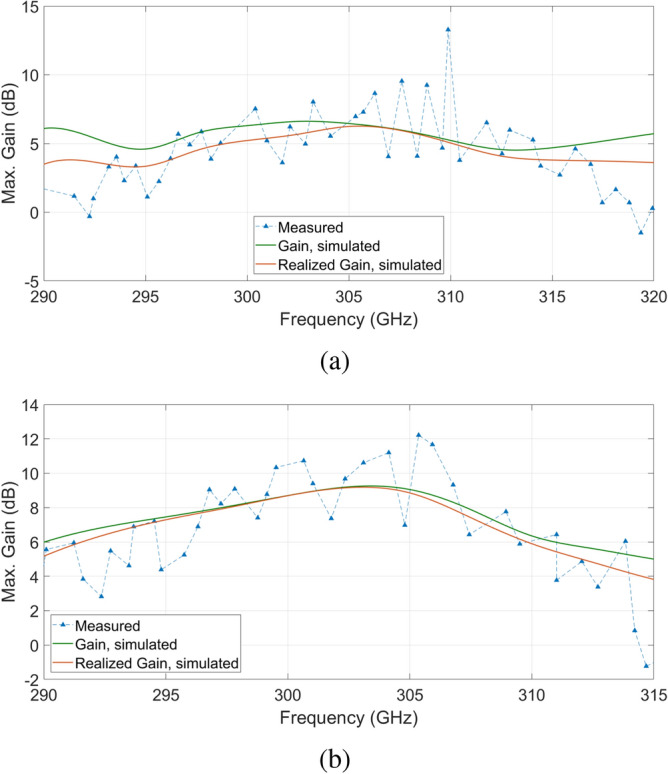
Measured and simulated gain of the non-MM-loaded and MM-loaded antennas over the frequency range of interest.

Figure 9 compares the simulated and measured maximum gain of the non-MM-loaded and the MM-loaded antennas over the bandwidth. A qualitative indication of how much the MM-cells change the electromagnetic dimension of the chip can be deduced from the trends of the simulated gain. By first looking at the non-MM-loaded case, a clear periodicity between the gain maximums of c.a. 15 GHz can easily be observed. By switching to the MM-loaded case such periodicity is lost, or rather stretched to higher frequency values. It is also worth to underline that the measured gain trends acceptably match the simulated ones.

For the sake of completeness, Table 1 reports the comparison between the proposed antennas and some of the most performing MM-based antenna found in the literature. In terms of the bandwidth and gain, the performance of the MM-loaded antenna is not so prominent. Yet the proposed MM-loaded antenna manages to achieve a 9-dB gain by using only less than 1.5 mm$^{2}$. It is proven to have the best aperture efficiency (which has been defined as $\lambda ^{2}\cdot 10^{Gain(dB)/10}/4\pi /Chip~dimension$).

**Table 1:** Comparison between the performances of the proposed antenna and the literature.

Ref.	Aperture eff.	Gain range (dBi)	Op. freq. (GHz)
This work	42.5%	6–9	297–313
^29^	1.16%	10–12.2	190–200
^30^	0.78%	7.58–8.56	350–385
^31^	0.62%	10.3–10.6	410–470
^32^	1.64%	9.6–11.6	290–316
^33^	36.4%	5.85–8.05	285–325

## Conclusion

In this paper, a gain enhancement of 2 dB and a beamwidth reduction of the main lobe have been observed on a sub-THz bow-tie antenna loaded with band-stop MM-cells. The latter provide an efficient surface wave suppression that has been achieved for the whole band of operation, leading to both a weakening of the undesired substrate modes and a better focus of the beam. Together with that, by comparing the measurements of three different samples, it appears that the MM-loaded antenna is less affected by the fabrication tolerances with respect to the non-MM-loaded one. The antenna has been designed with the IHPs 130 nm high performance SiGe BiCMOS BEOL metallization stack, and in order to maximize the overall performances, a portion of the Si-substrate has been removed under the antenna thanks to the LBE process. The MM-loaded antenna could represent a valid solution to overcome one of the main limitations when operating in the sub-THz range, e.g. the limited gain. Even though the gain enhancement brought by the MM-cells is not yet comparable to the one given by an antenna array or by a silicon lens, key advantages such as area minimization and simplicity in the implementation could make the difference in several commercial applications. In particular, an antenna array approach would be extremely troublesome to implement if to go for the LBE approach given the fact that the latter, as aforesaid, is only applicable to a very limited area, and having a series of cavities could seriously affect the yield of the test field. On the other side, a silicon lens doesn’t represent a feasible solution for those kind of applications where the cost is the principal key-factor.

## References

[1] Kleine-Ostmann T, Nagatsuma T (2011). A review on terahertz communications research. J. Infrared Millim. Terahertz Waves.

[2] Kallfass I, Boes F, Messinger T, Antes J, Inam A, Lewark U, Tessmann A, Henneberger R (2015). 64 Gbit/s transmission over 850 m fixed wireless link at 240 GHz carrier frequency. J. Infrared Millim. Terahertz Waves.

[3] Nagatsuma T, Ducournau GRC (2016). Advances in terahertz communications accelerated by photonics. Nat. Photon..

[4] Akyildiz I, Han C, Nie S (2018). Combating the distance problem in the millimeter wave and terahertz frequency bands. IEEE Commun. Mag..

[5] Petrov V, Kokkoniemi J, Moltchanov D, Lehtomaki J, Koucheryavy Y, Juntti M (2018). Last meter indoor terahertz wireless access: Performance insights and implementation roadmap. IEEE Commun. Mag..

[6] Chen Z, Chia M (2006). Broadband Planar Antennas.

[7] Balanis C (2015). Antenna Theory: Analysis and Design.

[8] Babakhani A, Guan X, Komijani A, Natarajan A, Hajimiri A (2006). A 77-GHz phased-array transceiver with on-chip antennas in silicon: Receiver and antennas. IEEE J. Solid State Circuits.

[9] Ng, H. J., Wang, R. & Kissinger, D. On-chip antennas in SiGe BiCMOS technology: Challenges, state of the art and future directions. In *2018 Asia-Pacific Microwave Conference (APMC)* (IEEE, Kyoto, Japan, 2018).

[10] Eldek AA, Elsherbeni AZ, Smith CE (2005). Wide-band modified printed bow-tie antenna with single and dual polarization for C-and X-band applications. IEEE Trans. Antennas Propag..

[11] Gutierrez F, Agarwal S, Parrish K, Rappaport TS (2009). On-chip integrated antenna structures in CMOS for 60 GHz WPAN systems. IEEE J. Sel. Areas Commun..

[12] Johannsen, U., Smolders, A. B., Mahmoudi, R. & Akkermans, J. A. G. Substrate loss reduction in antenna-on-chip design. In *2009 IEEE Antennas and Propagation Society International Symposium* (North Charleston, SC, USA, 2009).

[13] Chan KT, Chin A, Lin YD, Chang CY, Zhu CX, Li MF, Kwong DL, McAlister S, Duh DS, Lin WJ (2003). Integrated antennas on Si with over 100 GHz performance, fabricated using an optimized proton implantation process.. IEEE Microwave Wirel. Compon. Lett..

[14] Ng, H. J., Wessel, J., Genschow, D., Wang, R., Sun, Y. & Kissinger, D. Miniaturized 122 GHz system-on-chip radar sensor with on-chip antennas utilizing a novel antenna design approach. In *2016 IEEE MTT-S International Microwave Symposium (IMS)* (San Francisco, CA, USA, 2016).

[15] Zhang J, Ge X, Li Q, Guizani M, Zhang Y (2016). 5G millimeter-wave antenna array: Design and challenges. IEEE Wirel. Commun..

[16] Li Y, Luk KM (2014). Low-cost high-gain and broadband substrate-integrated-waveguide-fed patch antenna array for 60-GHz band.. IEEE Trans. Antennas Propag..

[17] Gao XJ, Cai T, Zhu L (2016). Enhancement of gain and directivity for microstrip antenna using negative permeability metamaterial. Int. J. Electron. Commun..

[18] Kim D, Choi J (2010). Analysis of antenna gain enhancement with a new planar metamaterial superstrate: An effective medium and a fabry-pérot resonance approach. J. Infrared, Millim. Terahertz Waves.

[19] Ravel F, Kosta YP, Joshi H (2015). Reduced size patch antenna using complementary split ring resonator as defected ground plane.. Int. J. Electron. Commun..

[20] Rajkumar R, Kiran KU (2016). A compact metamaterial multiband antenna for WLAN/WiMAX/ITU band applications. Int. J. Electron. Commun.(AEÜ).

[21] Khan, M. S., Tahir, F. A. & Cheema, H. M. Design of bowtie-slot on-chip antenna backed with E-shaped FSS at 94 GHz. In *2016 10th European Conference on Antennas and Propagation (EuCAP)* 1–3 (Davos, 2016).

[22] Wang, L. & Sun, W. Z. A 60-GHZ differential-fed circularly polarized on-chip antenna based on 0.18-m COMS technology with AMC structure. In *IET International Radar Conference 2015* 1–4 (Hangzhou,, 2015).

[23] Alibakhshikenari, M., Virdee, B. S., See, C. H., Abd-Alhameed, R. A., Falcone, F. & Limiti, E. *High-Gain Metasurface in Polyimide On-Chip Antenna Based on CRLH-TL for Sub-Terahertz Integrated Circuits Scientific Reports, 2020*. 10.1038/s41598-020-61099-8 PMC7062848 32152373

[24] Chen X, Grzegorczyk TM, Bae-Ian Wu, Pacheco J, Kong JA (2004). Robust method to retrieve the constitutive effective parameters of metamaterials. Phys. Rev. E.

[25] Gao, X. J., Cai, T. & Zhu, L. Enhancement of gain and directivity for microstrip antenna using negative permeability metamaterial. *Int. J. Electron. Commun.* (AEÜ) **70** (2016).

[26] Schmalz, K., Wang, R., Borngräber, J., Debski, W., Winkler, W. & Meliani, C. 245 GHz SiGe transmitter with integrated antenna and external PLL. In *2013 IEEE MTT-S International Microwave Symposium Digest (MTT)* (2013).

[27] Krzysztofik, W. J. & Cao, T. N. *Metamaterials in Application to Improve Antenna Parameters* (Metamaterials and Metasurfaces, Josep Canet-Ferrer, IntechOpen, 2018).

[28] Stocchi, M., Wietstruck, M., Schulze, S., Zhibo, C., Tolunay, S. & Kaynak, M. Full-wave RF modeling of a fan-out wafer-level packaging technology based on Al–Al wafer bonding. In *2020 IEEE 20th Topical Meeting on Silicon Monolithic Integrated Circuits in RF Systems, SiRF 2020* 60–62, 9040180 (2020).

[29] Alibakhshikenari M, Virdee BS, Salekzamankhani S (2021). High-isolation antenna array using SIW and realized with a graphene layer for sub-terahertz wireless applications. Sci. Rep..

[30] Alibakhshikenari M, Virdee BS, Althuwayb AA (2021). Study on on-chip antenna design based on metamaterial-inspired and substrate-integrated waveguide properties for millimetre-wave and THz integrated-circuit applications. J. Infrared. Milli Terahz Waves.

[31] Alibakhshikenari M, Virdee BS, Khalily M (2020). High-gain on-chip antenna design on silicon layer with aperture excitation for terahertz applications. IEEE Antennas Wirel. Propag. Lett..

[32] Alibakhshikenari M, Virdee BS, See CH (2020). Study on improvement of the performance parameters of a novel 0.41–0.47 THz on-chip antenna based on metasurface concept realized on 50 um GaAs-layer. Sci. Rep..

[33] Alibakhshikenari M, Virdee BS, See CH (2020). High-gain metasurface in polyimide on-chip antenna based on CRLH-TL for sub-terahertz integrated circuits. Sci. Rep..

